# Supplementary material for: Cascading effects of thermally-induced anemone bleaching on associated anemonefish hormonal stress response and reproduction
Source: Nat Commun. 2017 Oct 10;8:716. doi: 10.1038/s41467-017-00565-w (PMC5634421; doi:10.1038/s41467-017-00565-w)
Supplement: Supplementary file 1 — Supplementary Information [file 41467_2017_565_MOESM1_ESM.pdf]

Type of file: pdf

Title of file for HTML: Supplementary Information

Description: Supplementary Figures, Supplementary Tables

Type of file: pdf

Title of file for HTML: Peer Review File

Type of file: XLSX

Title of file for HTML: Supplementary Data 1

Description: Dependency of coastal fish species from French Polynesia to coral or anemone species.

Supplementary Figure 1. Location of anemone clusters and their bleaching status

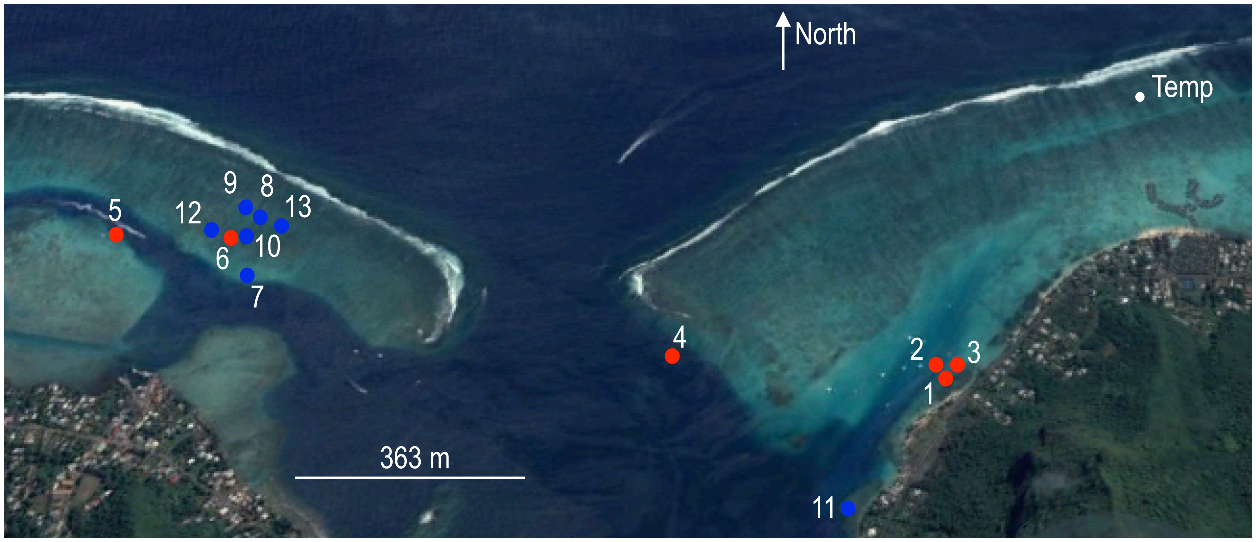

Distribution of the 7 unbleached (blue points) and 6 bleached (red points) anemone *Heteractis magnifica* locations with breeding *Amphiprion chrysopterus* pairs and the location of the bottom-mounted thermistors (Temp) by the MCR LTER[58] in the Northern lagoon of Moorea, French Polynesia monitored over 14 months from October 2015 through November 2016.

Supplementary Figure 2. Location of anemones with adjacent temperature thermistors and their bleaching status in 2017

A)

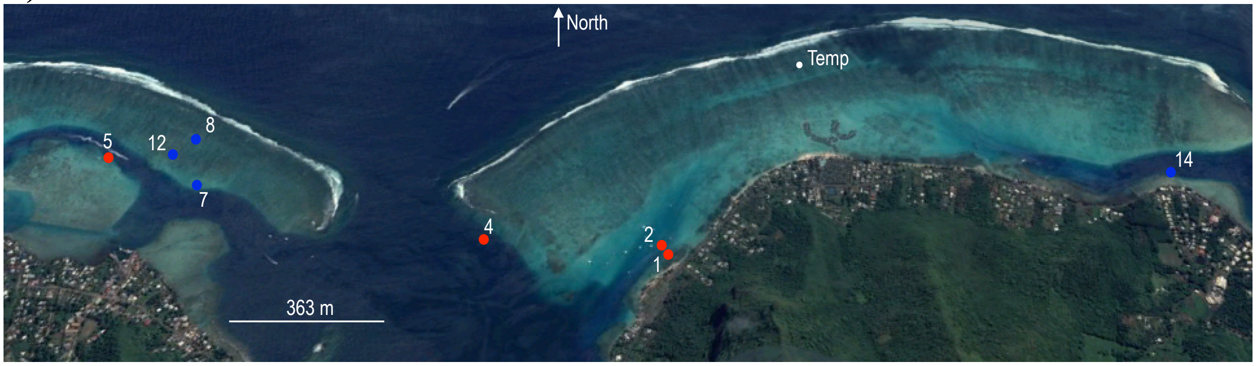

B) Mean maximum daily temperature

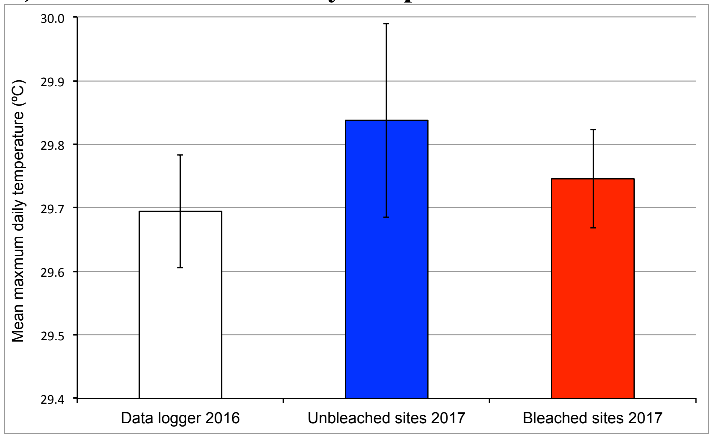

C) Mean daily temperature

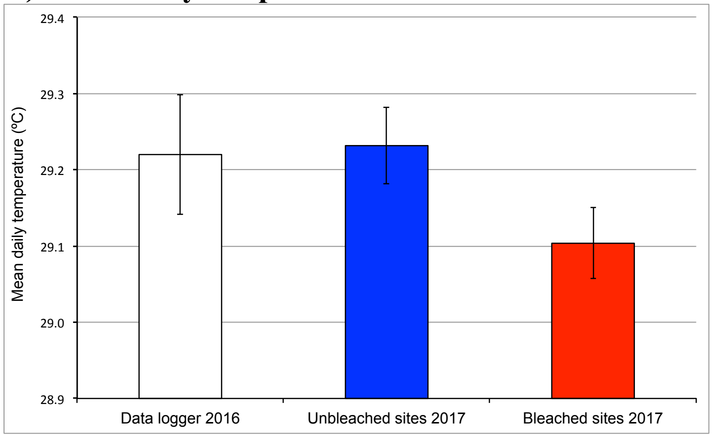

A) Distribution of the 4 unbleached (blue points) and 5 bleached (red points) anemones where 9 Marotte HS (High Sampling Rate), drag-tilt current meters (Marine Geophysics laboratory, James Cook University) were placed from March 21st through May 11th 2017, as well as the location of the bottom-mounted thermistors (Temp) by the MCR LTER[58], **B)** maximum and **C)** mean daily water temperatures over a similar period from March 21st through May 11th from the LTER in 2016 (white) and at unbleached (blue) and bleached (red) anemones in 2017.

Supplementary Figure 3. ***Amphiprion chrysopterus*** **embyronic development**

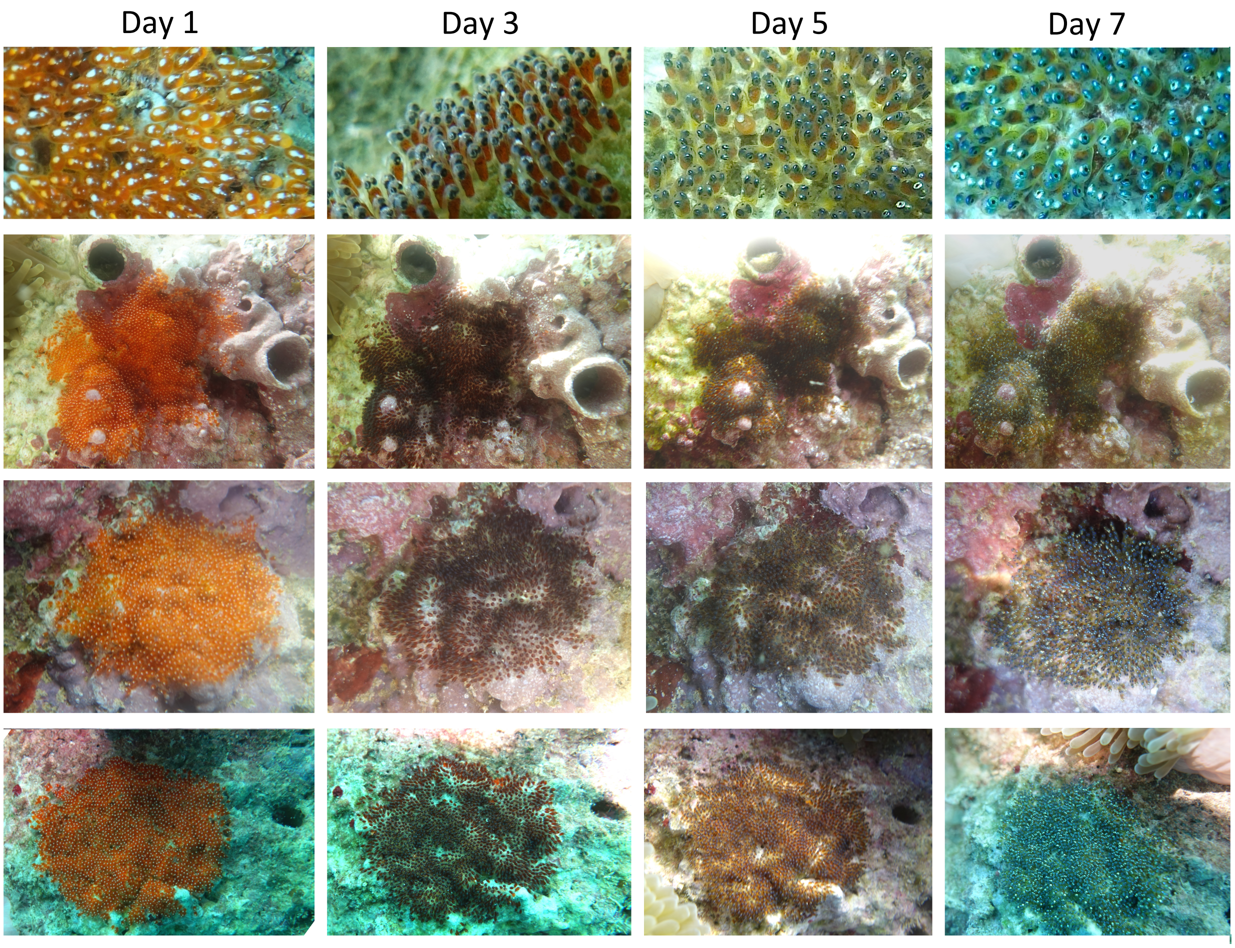

Embyronic development of wild, free-living *Amphiprion chrysopterus* eggs over 7 days in the Northern lagoon of Moorea, French Polynesia in 4 different nests.

Supplementary Figure 4. Validation of 17β estradiol EIA kit via parallelism

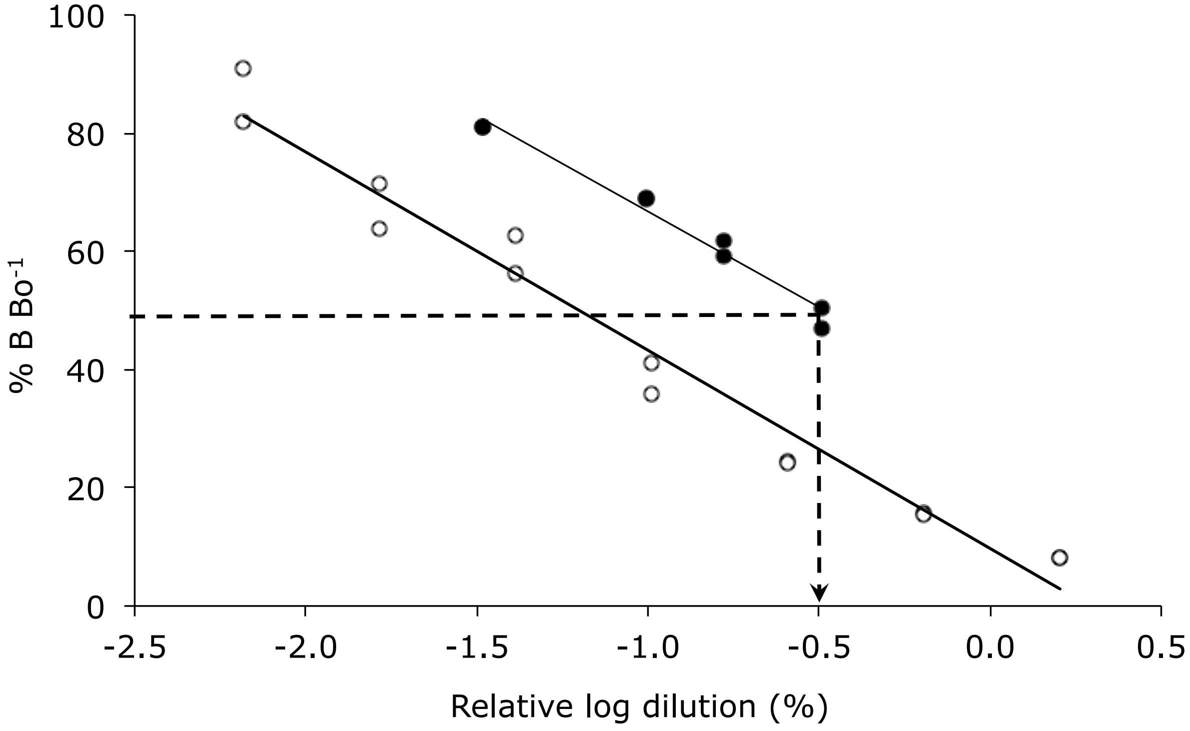

Dose-response curve for 17β estradiol obtained using 7 kit standards and pooled plasma from *Amphiprion chrysopterus* (kit standards: $y = -33.55\ x + 9.72$, $R^2 = 0.97$, N = 13, $p < 0.001$; samples: $y = -32.34\ x + 34.41$, $R^2 = 0.97$, N = 7, $p < 0.001$). Dashed line and arrow represents 50 % bound (see Supplementary Table 8 for corresponding dilution factors). ● = Pooled species plasma; ○ = 17β estradiol kit standards.

Supplementary Table 1. Statistical analyses of impacts of bleaching on endocrinology

A) Linear mixed model (LMM) of plasma hormone concentrations across sampling periods and bleaching status: April-May 2014 (no bleaching); February-March 2016, prior to the warming and bleaching event (no bleaching); and May 2016, during the peak anemone bleaching event from anemonefish pairs exposed to either bleached or unbleached anemones.

Hormone	Sex	*df*	*F*	*p*
Cortisol	**Females**	**3,41**	**7.309**	**< 0.001**
	**Males**	**3,47**	**3.464**	**0.023**
11-ketotestosterone	Females	3,38	0.077	0.972
	**Males**	**3,34**	**4.460**	**0.010**
17β estradiol	**Females**	**3,27**	**7.840**	**0.001**
	Males	3,23	2.986	0.052

df, degrees of freedom; *F*, test statistic: *p*, probability.

B) Estimates of variance parameters in the above LMM which indicate that a random effect is only needed in the tests for 11-ketotestoterone in the case of females and for estradiol in the case of males.

Hormone	Sex	*Estimate ± SE*	*Wald Z*	*p*
Cortisol	Females	0.027 ± 0.08	0.332	0.740
	**Males**	0.004 ± 0.02	0.187	0.852
11-ketotestosterone	**Females**	**0.00008 ± 0.00002**	**3.687**	**<0.001**
	Males	0.00007 ± 0.00007	0.947	0.344
17β estradiol	Females	47.841 ± 51.93	0.921	0.357
	**Males**	**9.228 ± 3.97**	**2.324**	**0.020**

Estimate, estimate of variance parameters; *Wald Z*, test statistic: *p*, probability.

Supplementary Table 2. Statistical analyses of impacts of bleaching on plasma cortisol levels

Bonferroni pairwise comparisons of fixed effects (sampling period / bleaching status) from linear mixed model of plasma cortisol concentrations: April-May 2014; February-March 2016, prior to the bleaching event; and May 2016, during the anemone bleaching event from anemonefish pairs exposed to either bleached or unbleached anemones.

During the bleaching event, cortisol levels in both sexes were elevated in anemonefish exposed to bleached compared to unbleached hosts. Furthermore, female anemonefish exposed to bleached hosts had higher cortisol levels compared to those sampled prior to the bleaching event in 2016, and anemonefish of both sexes exposed to bleached hosts had higher cortisol levels compared to those sampled in 2014. On the other hand, there were no differences in cortisol levels from anemonefish exposed to unbleached hosts during the bleaching event compared to either those sampled prior to the bleaching event in 2016, or compared to those sampled in 2014, nor between samples taken in 2014 and 2016.

Hormone	Sex	Bonferroni pairwise comparisons	*df*	Mean	*p*
Cortisol	Females	2014-2016 prior	2,38	0.562	1.000
		2014-2016 unbleached	2,38	5.421	1.000
		**2014-2016 bleached**	**2,38**	**22.193**	**<0.001**
		2016 prior-2016 unbleached	2,38	4.860	1.000
		**2016 prior-2016 bleached**	**2,38**	**21.632**	**0.004**
		**2016 unbleached-2016 bleached**	**2,38**	**16.772**	**0.024**
Cortisol	Males	2014-2016 prior	2,44	2.036	1.000
		2014-2016 unbleached	2,44	1.299	1.000
		**2014-2016 bleached**	**2,44**	**13.515**	**0.035**
		2016 prior-2016 unbleached	2,44	3.335	1.000
		2016 prior-2016 bleached	2,44	11.479	0.348
		**2016 unbleached-2016 bleached**	**2,44**	**14.814**	**0.051**

df, degrees of freedom; *F*, test statistic; *p*, probability; adjustment for multiple comparisons: Bonferroni

Supplementary Table 3. Statistical analyses of impacts of bleaching on plasma 11-ketotestosterone levels

Bonferroni pairwise comparisons of fixed effects (sampling period / bleaching status) from linear mixed model of plasma 11-ketotestosterone concentrations: April-May 2014; February-March 2016, prior to the bleaching event; and May 2016, during the anemone bleaching event from anemonefish pairs exposed to either bleached or unbleached anemones.

11-KT levels showed a non-significant trend to be lower in male anemonefish exposed to bleached compared to unbleached hosts, but not in females. Furthermore, 11-KT levels were lower in male anemonefish exposed to bleached hosts compared to those prior to the bleaching event in 2016, but not females, and compared to those taken in 2014, but not females. On the other hand, there were no differences in 11-KT levels from either male or female anemonefish exposed to unbleached hosts during the bleaching event compared to either those prior to the bleaching event in 2016, to those taken in 2014, nor between samples taken in 2014 and 2016.

Hormone	Sex	Bonferroni pairwise comparisons	*df*	Mean	*p*
11-ketotestosterone	Females	2014-2016 prior	2,38	0.014	1.000
		2014-2016 unbleached	2,38	0.001	1.000
		2014-2016 bleached	2,38	0.020	1.000
		2016 prior-2016 unbleached	2,38	0.015	1.000
		2016 prior-2016 bleached	2,38	0.006	1.000
		2016 unbleached-2016 bleached	2,38	0.021	1.000
11-ketotestosterone	Males	2014-2016 prior	2,33	0.076	1.000
		2014-2016 unbleached	2,33	0.012	1.000
		**2014-2016 bleached**	**2,33**	**0.286**	**0.032**
		2016 prior-2016 unbleached	2,33	0.088	1.000
		**2016 prior-2016 bleached**	**2,33**	**0.362**	**0.017**
		2016 unbleached-2016 bleached	2,33	0.274	0.071

df, degrees of freedom; *F*, test statistic; *p*, probability; adjustment for multiple comparisons: Bonferroni

Supplementary Table 4. Statistical analyses of impacts of bleaching on plasma 17β estradiol levels

Bonferroni pairwise comparisons of fixed effects (sampling period / bleaching status) from linear mixed model of plasma 17β estradiol concentrations: April-May 2014; February-March 2016, prior to the bleaching event; and May 2016, during the anemone bleaching event from anemonefish pairs exposed to either bleached or unbleached anemones.

During the bleaching event, estradiol levels were lower in female anemonefish exposed to bleached compared to unbleached hosts, but not males. Furthermore, 17β-estradiol levels were also lower in female anemonefish exposed to bleached hosts compared to those both prior to the bleaching event in 2016, but not males, and in 2014, but not males. On the other hand, there were no differences in 17β-estradiol levels from either male or female anemonefish exposed to unbleached hosts prior to the bleaching event in 2016 compared to those during.

Hormone	Sex	Bonferroni pairwise comparisons	*df*	Mean	*p*
17β estradiol	Females	2014-2016 prior	2,27	221.085	0.401
		2014-2016 unbleached	2,27	193.906	0.632
		**2014-2016 bleached**	**2,27**	**524.040**	**<0.001**
		2016 prior-2016 unbleached	2,27	27.179	1.000
		**2016 prior-2016 bleached**	**2,27**	**302.955**	**0.043**
		**2016 unbleached-2016 bleached**	**2,27**	**303.134**	**0.023**
17β estradiol	Males	2014-2016 prior	2,44	109.555	0.058
		2014-2016 unbleached	2,44	91.121	0.194
		2014-2016 bleached	2,44	104.882	0.067
		2016 prior-2016 unbleached	2,44	18.435	1.000
		2016 prior-2016 bleached	2,44	4.673	1.000
		2016 unbleached-2016 bleached	2,44	13.761	1.000

df, degrees of freedom; *F*, test statistic; *p*, probability; adjustment for multiple comparisons: Bonferroni

Supplementary Table 5. Statistical analyses of impacts of bleaching on reproductive function

General linear model with repeated measures of anemonefish reproductive function (number of eggs laid and number of viable pre-hatching eggs) over 10 months from October 2015 through July 2016.

Reproductive function	Sex	*df*	*Mean square*	*F*	*p*
Number of eggs laid	**Month**	**1,9**	**8978930**	**27.746**	**<0.001**
	Bleaching	1,9	1318438	1.211	0.295
	**Month*bleaching**	**1,9**	**4217241**	**13.032**	**0.004**
Viable eggs at hatching	**Month**	**1,9**	**3742717**	**20.261**	**0.001**
	bleaching	1,9	1523808	1.723	0.216
	**Month*bleaching**	**1,9**	**1595946**	**8.640**	**0.013**

df, degrees of freedom; *F*, test statistic: *p*, probability.

Supplementary Table 6. Statistical analyses of impacts of bleaching on reproductive function

General linear model with repeated measures of anemonefish reproductive function (number of eggs laid and number of viable pre-hatching eggs) split into three periods: 5 months prior to the bleaching event from October 2015 through February 2016, 5 months during the bleaching event from March 2016 through July 2016 and 4 months after the bleaching even from August 2016 through November 2016.

Reproductive function	Period	Sex	*df*	*Mean square*	*F*	*p*
Number of eggs laid	Before	**Month**	**1,9**	**1166424**	**7.471**	**0.019**
		Bleaching	1,9	848	0.001	0.971
		Month*bleaching	1,9	8399	0.054	0.821
	During	Month	1,9	167826	0.763	0.401
		**Bleaching**	**1,9**	**2946760**	**6.927**	**0.023**
		Month*bleaching	1,9	306750	1.395	0.262
	After	Month	1,9	124266	1.790	0.211
		Bleaching	1,9	29438	0.045	0.837
		Month*bleaching	1,9	45423	0.654	0.437
Viable eggs at hatching	Before	**Month**	**1,9**	**2957100**	**7.464**	**0.020**
		Bleaching	1,9	197854	0.249	0.628
		Month*bleaching	1,9	99140	0.250	0.627
	During	Month	1,9	85806	0.150	0.706
		**Bleaching**	**1,9**	**4279334**	**6.397**	**0.028**
		Month*bleaching	1,9	1300536	2.279	0.159
	After	Month	1,9	13558	0.038	0.850
		Bleaching	1,9	312639	0.432	0.526
		Month*bleaching	1,9	193037	0.535	0.481

df, degrees of freedom; *F*, test statistic: *p*, probability.

Supplementary Table 7. Statistical analyses of impacts of bleaching on spawning frequency

Cochran's Q test General linear model with repeated measures of anemonefish reproductive function (spawning frequency) over 14 months from October 2015 through November 2016.

Reproductive function	Bleaching group	*df*	*Mean square*	*Q*	*p*
Spawning frequency	Unbleached	1,13	0.132	14.757	0.323
	**Bleached**	**1,13**	**0.462**	**28.000**	**0.009**

df, degrees of freedom; *F*, test statistic: *p*, probability.

Supplementary Table 8. Statistical analyses of impacts of bleaching on reproductive function

Paired *t*-tests (Total number of eggs laid and Number of viable pre-hatching eggs) or Wilcoxon Signed Ranks Test (Mortality and Spawning frequency) of anemonefish exposed to unbleached or bleached anemones during the bleaching event between three periods: before the bleaching event (October 2015 through February 2016), during bleaching (March through July 2016) and after the bleaching event (August through November 2016).

Reproductive function	Bleaching	Sex	*n*	*t or Z*	*p*
Number of eggs laid on Day 1/2	Unbleached	Before - During	7	1.962	0.097
		Before - After	7	0.636	0.549
		During - After	7	1.695	0.141
	Bleached	**Before - During**	**6**	**4.965**	**0.004**
		Before - After	6	1.040	0.346
		**During - After**	**6**	**5.205**	**0.003**
Viable eggs at hatching on Day 6/7	Unbleached	Before - During	7	1.445	0.199
		Before - After	7	0.294	0.779
		During - After	7	1.188	0.280
	Bleached	**Before - During**	**6**	**6.585**	**0.001**
		Before - After	6	0.613	0.567
		**During - After**	**6**	**3.897**	**0.011**
Mortality Δ Day 1/2 - Day 6/7	Unbleached	Before - During	7	0.169	0.866
		Before - After	7	0.169	0.866
		During - After	7	1.183	0.237
	Bleached	**Before - During**	**6**	**1.992**	**0.046**
		Before - After	6	0.105	0.917
		**During - After**	**6**	**1.992**	**0.046**
Spawning frequency	Unbleached	Before - During	7	1.342	0.180
		Before - After	7	1.105	0.269
		During - After	7	1.761	0.078
	Bleached	**Before - During**	**6**	**2.003**	**0.045**
		Before - After	6	1.069	0.285
		**During - After**	**6**	**2.207**	**0.027**

n, total number of paired samples; *t*, test statistic for paired t-test; Z, test statistic for Wilcoxon Signed Ranks Test; *p*, probability.

Supplementary Table 9. Water temperature differences between sites with bleached and unbleached anemones in 2017

A) General linear model of water temperature at anemone sites that were bleached and unbleached in 2017 compared to those recorded from the long-term logger for the same period in 2016 (March 21st through May 11th) and **B)** their simple contrasts.

Temperature °C	Group	*Mean square*	*F*	*p*
Maximum	2016 / 2017	0.301	0.048	0.954
	**Anemone site**	**3.668**	**5.681**	**<0.001**
Average	2016 / 2017	0.291	0.110	0.897
	**Anemone site**	**1.486**	**9.538**	**<0.001**

F, test statistic: *p*, probability.

B) Simple contrasts from GLM of water temperature at anemone sites that were bleached and unbleached in 2017 compared to those recorded from the long-term logger for the same period in 2016 (March 21st through May 11th).

Temperature °C	Group	*Contrast*	*p*
	2017 unbleached vs 2017 bleached	0.087	0.528
Maximum	2017 unbleached vs 2016 logger	0.144	0.344
	2017 bleached vs 2016 logger	0.057	0.694
	2017 unbleached vs 2017 bleached	0.117	0.086
Average	2017 unbleached vs 2016 logger	0.010	0.896
	2017 bleached vs 2016 logger	0.107	0.132

p, probability.

Supplementary Table 10. Validation of the 17β estradiol EIA kit in *Amphiprion chrysopterus*

ANCOVA on homogeneity of slopes for sample dilution *versus* standard dilution curves for 17β estradiol kits in *Amphiprion chrysopterus*. The dilution factor (dilution) for 50 % of antibody bound determined from a regression analysis is also given.

Assay	Species	*df*	Mean square	*F*	*p*	Dilution factor
17β estradiol	*A. chrysopterus*	1,21	0.001	0.089	0.769	1:3 (0.33)

df, degrees of freedom; *F*, test statistic: *p*, probability.
